# Supplementary figures and images for: Physioxia Has a Beneficial Effect on Cartilage Matrix Production in Interleukin-1 Beta-Inhibited Mesenchymal Stem Cell Chondrogenesis
Source: Cells. 2019 Aug 20;8(8):936. doi: 10.3390/cells8080936 (PMC6721827; doi:10.3390/cells8080936)

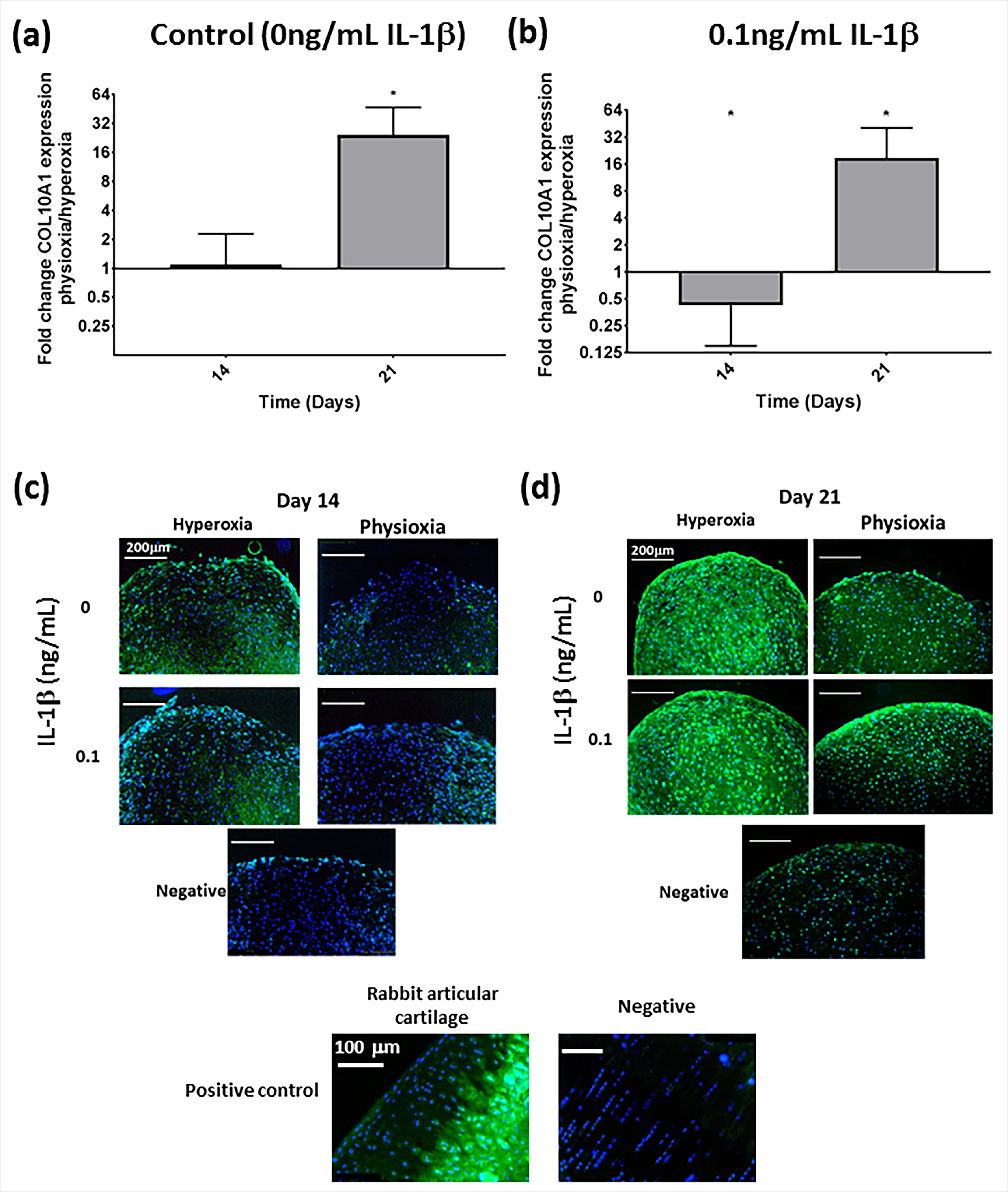

Supplement: Supplementary file 1 [file cells-08-00936-s001.zip › cells-489057-supplementary.tif]
